# Decoding dysphoria and violence: phenomenological insights to diagnosis and therapeutic interventions

**DOI:** 10.1192/j.eurpsy.2025.181

**Published:** 2025-08-26

**Authors:** L. Madeira

**Affiliations:** Universidade de Lisboa, Faculdade de Medicina, Lisboa, Portugal

## Abstract

**Abstract:**

Aggressive emotions—such as anger, rage, envy, and resentment—play a complex role in human experience, spanning personal, social, and psychopathological dimensions. While anger can serve as a protective, communicative, or motivational force, it can also escalate into destructive emotions such as revenge, hatred, or resentment. This talk explores the phenomenology, psychology, and psychopathology of aggressive emotions, drawing from philosophical, psychoanalytic, and neuroscientific perspectives. These have a bodily-affective nature, shaping perception, behavior, and interpersonal dynamics (Schmitz, 2019; Landweer, 2020). While anger is often a reaction to a perceived transgression, it also functions as a regulatory mechanism for social norms and personal boundaries (Berkowitz, 1962; Bandura, 1973). I examine the ontological independence of emotions, showing how their spatial and embodied qualities influence their regulation and transformation (Fuchs, 2005). A key focus will be the transformation of anger into resentment and revenge, using Nietzsche and Scheler’s theories of ressentiment to explore how powerlessness fuels hostility (Nietzsche, 1887; Scheler, 1912). We discuss how pathological resentment differs from normative anger, leading to chronic hostility, ideological fixation, and moral superiority complexes. Finally, I analyze the role of aggressive emotions in psychopathology, considering their manifestations in personality disorders, trauma-related disorders, and psychotic states (Novaco, 1979; Cameron, 1943). From paranoia’s persecutory anger to the dysregulated aggression of borderline and antisocial personalities, the keynote explores how anger-related emotions are structured, experienced, and acted upon across clinical categories.

**Disclosure of Interest:**

None Declared

